# Comparison of two types of platelet-rich plasma in rotator cuff injury: study protocol for a randomized clinical trial

**DOI:** 10.3389/fmed.2026.1791881

**Published:** 2026-03-13

**Authors:** Yuxiu Ji, Bo Wang, Shengjian Wu, Xiaoqiao Chen, Yujie Xie, Xi Luo, Chi Zhang, Li Wang

**Affiliations:** 1 Rehabilitation Medicine Department, The Affiliated Hospital of Southwest Medical University, Luzhou, Sichuan, China; 2 Rehabilitation Medicine and Engineering Key Laboratory of Luzhou, Department of Rehabilitation Medicine, Southwest Medical University, Luzhou, Sichuan, China; 3 Department of Rehabilitation Medicine, Southwest Medical University, Luzhou, Sichuan, China

**Keywords:** leukocyte, platelet-rich plasma, protocol, PRP, rotator cuff injury, tendinopathy

## Abstract

**Background:**

Platelet-rich plasma (PRP) has been widely used in the treatment of rotator cuff tendinopathy. Due to the lack of unified standards for PRP preparation and clinical instructions, its efficacy remains controversial.

**Purpose:**

We aimed to conduct a pragmatic comparison of two commonly used composite PRP formulations with different platelet (PLT) and leukocyte concentrations to provide clinical evidence for the optimal PRP formulation in the treatment of rotator cuff injury (RCI).

**Methods:**

A prospective, single-center, participant-blinded, outcome-assessor-blinded, and statistician-blinded randomized controlled trial with a 12-month follow-up investigating PRP in RCI will be conducted at Southwest Medicine University. Participants who choose PRP treatment will be randomly allocated in a 1:1 ratio to either group. Clinical assessments will be performed at baseline (2 days before PRP injection) and 1, 3, 6, and 12 months after the first injection. The primary outcomes include the Chinese version of the Constant-Murley Score (CMS) and the visual analog scale (VAS). Secondary outcomes include imaging changes assessed by magnetic resonance imaging (MRI) and ultrasound (US) scanning, serum cytokine measurements, the 12-Item Short Form (SF-12), and adverse events (AEs). Statistical analysis will be performed according to the intention-to-treat (ITT) principle.

**Implications:**

The results will provide new evidence for the optimal PRP formulation for RCI in clinical practice. Furthermore, we will investigate multiple outcomes, including clinical, functional, structural, and inflammatory changes after PRP injection.

**Trial registration:**

https://www.chictr.org.cn/searchproj.html?title=&officialname=&subjectid=&regstatus=&regno=ChiCTR2500113374&secondaryid=&applier=&studyleader=&createyear=&sponsor=&secsponsor=&sourceofspends=&studyailment=&studyailmentcode=&studytype=&studystage=&studydesign=&recruitmentstatus=&gender=&agreetosign=&measure=&country=&province=&city=&institution=&institutionlevel=&intercode=&ethicalcommitteesanction=&whetherpublic=&minstudyexecutetime=&maxstudyexecutetime=&btngo=btn, This study was registered in the Chinese clinical trial registry (ChiCTR2500113374).

## 1 Introduction

Rotator cuff injury (RCI) is a prevalent musculoskeletal disorder, with prevalence ranging from 5 to 39%, leading to intense shoulder pain, aberrant shoulder movement, and progressive muscle atrophy (1). When selecting the optimal treatment for patients with symptomatic RCI, clinicians should take tear size, shoulder function and stability, and patient preferences into full consideration (2). Currently, with the rapid development of arthroscopy, surgical repair can relieve pressure on the injured rotator cuff; however, postoperative pain and tendon-bone healing outcomes remain variable (1, 2). The focus of the current trend has shifted toward biological enhancement rather than simple mechanical repair (3). The clinical application of various biological agents, such as platelet-rich plasma (PRP), stem cells, or purified exosomes, has been widely explored in the treatment of tendinopathy (4–7).

PRP, an autologous blood product containing a high concentration of platelets (PLTs), has been widely used in RCI (8). Once PLTs in PRP are activated, p-granules undergo degranulation and then release growth factors (GFs) and cytokines that can modulate the microenvironment (9). With supraphysiological concentrations of PLTs, pathological tissues can mimic the initial stages of healing to rapidly enhance repair signals during the inflammatory phase (10). In addition, GFs can promote vascularization and stimulate angiogenesis along with cell proliferation (10–13).

Theoretically, PRP can promote rotator cuff tendon healing. However, controversies surrounding its efficacy may arise from variables throughout the process—from manufacturing to clinical application—that can affect PRP quality and effectiveness (4, 14), such as preparation methods, PLT concentration, and cellular composition (15). The relationship between platelet concentration and its biological effect is not linear. For any given tissue, there exists a therapeutic window; concentrations below this threshold may be insufficient to stimulate a robust healing response, while concentrations above it could potentially inhibit cellular activity or promote an excessive inflammatory reaction, thereby hindering repair. For tendinopathy, the optimal PLT concentration is 3–5 times that of the physiological concentration in the whole blood (16). Beyond PLT concentration, the role of leukocytes in PRP is also controversial (17). Leukocyte-rich PRP (LR-PRP) can aggravate the inflammatory response, particularly due to pro-decomposition factors produced by neutrophils, which can accelerate the apoptosis of tendon cells and chondrocytes (18, 19). Leukocyte-poor PRP (LP-PRP), which can promote collagen formation and reduce the inflammatory environment, may be particularly suitable for acute RCI (3). Liu et al. (15) proposed that the biological status of the tendon, rather than leukocyte concentration, ultimately determines the efficacy of tendon healing.

For tendon repair, the goal is to restore the organized structure and reduce the tear rate by promoting cellular differentiation. However, which type of PRP is optimal for the treatment of RCI remains uncertain. It is essential to assess the components of PRP before injection into the injured sites and to understand the roles of different cytokines and how they relate to improvements in clinical outcomes. In this study, we aim to perform a pragmatic clinical comparison of two composite PRP formulations—low-PLT LP-PRP and high-PLT LR-PRP—with different PLT and leukocyte concentrations, both of which are widely used in clinical practice. We intend to explore the clinical efficacy of these two formulations and provide evidence for the optimal PRP formulation for RCI.

## 2 Materials and methods

### 2.1 Study design, randomization, and blinding

A prospective, single-center, participant-blinded, outcome-assessor-blinded, and statistician-blinded randomized controlled trial was scheduled to commence in January 2026, with a 12-month follow-up at the Affiliated Hospital of Southwest Medical University. The primary endpoint is set at 12 months after the first injection, allowing assessment of functional, structural, and inflammatory recovery. This study strictly complies with the Declaration of Helsinki and involves human participants. The study design adheres to the SPIRIT statement and is registered in the Chinese Clinical Trial Registry (ChiCTR2500113374), with ethical approval obtained from the Institutional Review Board (IRB) of the Affiliated Hospital of Southwest Medical University (KY2025570). All participants will sign a written informed consent form before enrollment, which clearly states the study purpose, intervention measures, follow-up plan, potential risks and benefits, and the right to withdraw from the study at any time without affecting clinical care.

After recruitment and baseline assessment, eligible patients will be randomized in a 1:1 ratio into two groups (group 1: low-PLT LP-PRP; group 2: high-PLT LR-PRP). Stratified randomization will be performed based on age (18–45 years old vs. 46–65 years old), Ellman classification (Grade I/II vs. Grade III), and the injury-to-injection interval (0–2 weeks vs. 2–4 weeks) to ensure the balance of key confounding factors between the two groups. The random sequence will be generated by an independent researcher (BW) and stored in sealed envelopes. The envelopes will be opened by BW only to obtain the group code before PRP preparation, ensuring strict allocation concealment. PRP will be prepared by XQC, which is independent of patient care and outcome assessment. XQC will only receive the group code and will not contact patients or participate in any clinical evaluation processes. The prepared PRP will be loaded into uniform, unmarked sterile syringes, and the injection physician (YJX), who performs ultrasound (US)-guided injections, will only carry out the injection procedure and remain blinded to group allocation. All patients and outcome assessors (SJW and XL) will also be blinded to treatment allocation, and YXJ will remain blinded during data analysis and will be unblinded only after all statistical analyses are completed. An independent, non-blinded researcher (LW) will be responsible for trial safety monitoring and maintaining allocation concealment. LW is authorized to unblind individual patients only in the event of serious adverse events (SAEs) and will not participate in patient diagnosis, treatment, outcome assessment, or data analysis. CZ will monitor the entire trial process to ensure the feasibility and accuracy of the study.

### 2.2 Participant enrollment

Patient recruitment will be conducted from January 2026 to December 2026, with a planned recruitment period of 12 months, at the Affiliated Hospital of Southwest Medical University. The inclusion criteria are as follows (20–22): (1) age between 18 and 65 years; (2) patients presenting with clinical signs of shoulder pain and dysfunction who are not candidates for surgery; (3) severity of RCI according to the Ellman classification system: grade I (<3 mm, <25% tendon thickness), grade II (3–6 mm, 25–50% tendon thickness), and grade III (>6 mm, >50% tendon thickness) as determined by magnetic resonance imaging (MRI), with a disease course of 2–6 weeks (subacute stage); (4) hemodynamic stability; (5) patients willing to undergo PRP injection therapy; and (6) patients who provide informed consent and can comply with all study procedures and requirements.

The exclusion criteria are as follows (23): (1) age <18 years or >65 years; (2) previous scapula dysfunction or shoulder joint activity disorders resulting from central or peripheral nerve injury; (3) external injury combined with an upper limb fracture; (4) patients who have undergone prior surgical intervention or plan to undergo surgical repair within 6 months; (5) patients who have received shoulder injection therapy, such as steroid injection, within 6 months; (6) patients with blood disorders, including PLT dysfunction syndrome, PLT count <100,000/μL, or hemoglobin <10 g/dL; (7) patients with fever or potential infectious disease within 2 weeks; (8) patients with systemic diseases, a history of cancer, long-term use of antiplatelet agents, habitual analgesic use, or other conditions that make them unsuitable for rehabilitation therapy or PRP injection; and (9) patients who have participated in other drug studies within 30 days.

**Figure 1:**
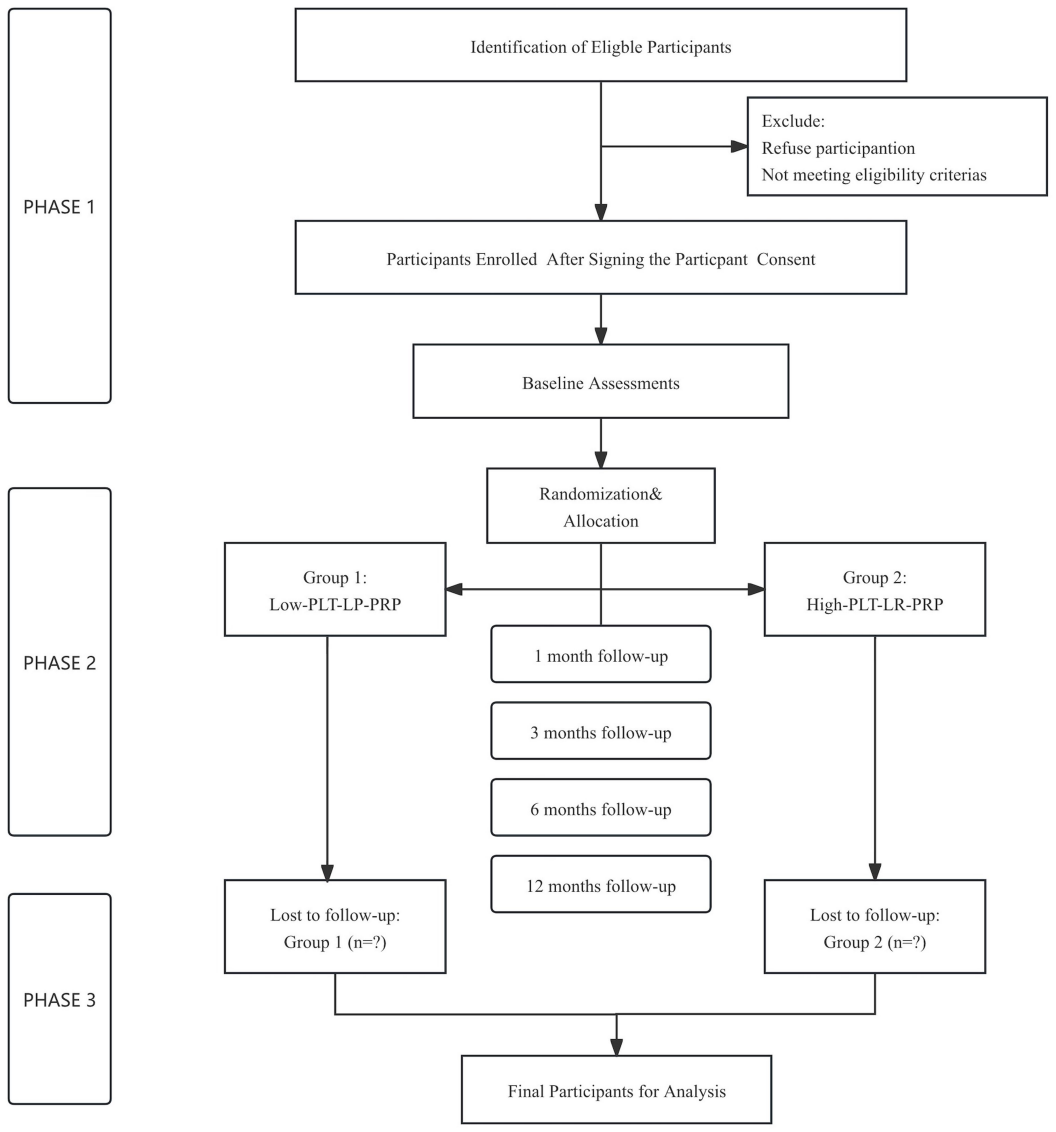
Flowchart of the overall study design.

The trial protocol is shown in Figure 1.

### 2.3 Procedures

#### 2.3.1 PRP intervention and basic characteristics

Clinically, two types of PRP are commonly used: Composite PRP formulations characterized by either a lower PLT and leukocyte concentration or a higher PLT and leukocyte concentration. The classification of PRP follows previous studies (24, 25). All included participants will receive one of the two types of PRP injections, administered randomly once a week for a total of 2 weeks.

The final PRP volume for each injection session is 2 mL. The absolute PLT count administered to each patient will be calculated by multiplying the PLT concentration measured in the final PRP product (×10^9^/L) by the injection volume (2 mL). Blood cell counts of whole blood and PRP will be analyzed using an automated hematology analyzer. PLT concentrations and absolute counts for both groups are presented in Table 1. All values will be recorded in case report forms (CRFs) for subsequent correlation analyses with clinical outcomes.

**Table 1:** Characteristics of PRP formulations and absolute platelet counts.

Group	WBC (×10^9^/L)	NEU (×10^9^/L)	RBC (×10^12^/L)	PLT (×10^9^/L)	Absolute PLT count (×10^9^/2 mL)	PLT enrichment (fold)
Group 1	0.11 ± 0.11	0	0	868.67 ± 98.51	1.74 ± 0.20	2.5–3.5
Group 2	29.29 ± 0.56	15.49 ± 1.41	0.16 ± 0.16	1329.00 ± 261.00	2.66 ± 0.52	4.0–6.0

Data are presented as mean ± standard deviation.

RBC, red blood cell; WBC, white blood cell; NEU, neutrophil; PLT, platelet.

The absolute platelet count was calculated based on a 2 mL injection volume.

PLT enrichment fold was estimated relative to the mean whole blood platelet concentration (200–300 × 10^9^/L).

#### 2.3.2 Standardization of injection timing

The timing of PRP injection, from the diagnosis of RCI to the first injection, will be standardized for all participants. The injury occurrence time is defined as the exact date when shoulder pain and motor dysfunction first appeared. For patients with an insidious onset, the injury occurrence time is defined as the date of MRI confirmation of RCI. The injection date will be recorded on the exact day to calculate the injury-to-injection time interval (days). For patients with subacute RCI (symptom duration 2–6 weeks), those with an injury-to-injection time interval of 0–2 weeks are included in the first subgroup, while those with a 2–4 week interval are included in the second subgroup, ensuring a balanced distribution between the two groups. The mean injury duration and injury-to-injection time interval will be recorded at baseline and compared between the groups. If significant differences are detected, these variables will be included as covariates in statistical analysis.

#### 2.3.3 PRP preparation

**Figure 2:**
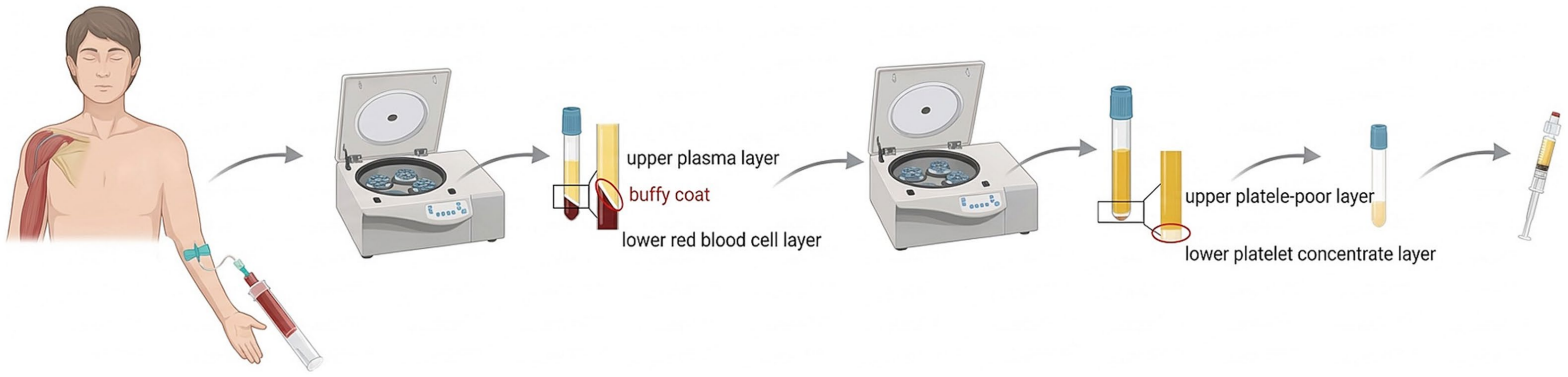
Preparation of the two types of PRP.. Created with BioRender.com.

PRP products will be prepared according to the manufacturer’s instructions for the device (JZK-8, China) using a double centrifugation protocol (Figure 2). All centrifugation procedures will be performed at 25 °C to ensure the stability of PLT and leukocyte activity. Peripheral blood (20 mL) will be collected from patients into a tube containing an anticoagulant (acid citrate sodium citrate solution) under sterile conditions. Subsequently, the anticoagulant will be gently mixed with the whole blood before transferring it to centrifuge tubes. The PRP preparation method will be chosen according to the allocated group. Finally, 2 mL of PRP will be collected for clinical application in patients with RCI.

##### 2.3.3.1 Group1: low-PLT LP-PRP

The entire tube will be placed into a secondary device supplied by the same manufacturer for the first centrifugation at 1200 rpm for 10 min, and three layers—the upper plasma layer, the middle buffy coat layer, and the lower red blood cell layer—will be observed in this process. To minimize the leukocyte effect, only the upper 80% of the plasma layer will be drawn and transferred to a new tube, avoiding the buffy coat and red blood cell layers. The plasma and PLT will be re-centrifuged at 3000 rpm for 15 min to maintain the bottom PLT layer, ultimately yielding approximately 2 mL of PRP.

##### 2.3.3.2 Group 2: high-PLT LR-PRP

The first centrifugation will be performed at 1500 rpm for 10 min with three layers. The plasma and PLT, including the buffy coat, will be drawn into a new sterile tube and re-centrifuged at 2500 rpm for 10 min. Two-thirds of the upper platelet-poor plasma (PPP) will then be removed, retaining the bottom PLT concentrate to yield approximately 2 mL of PRP.

#### 2.3.4 Injection procedure

**Figure 3:**
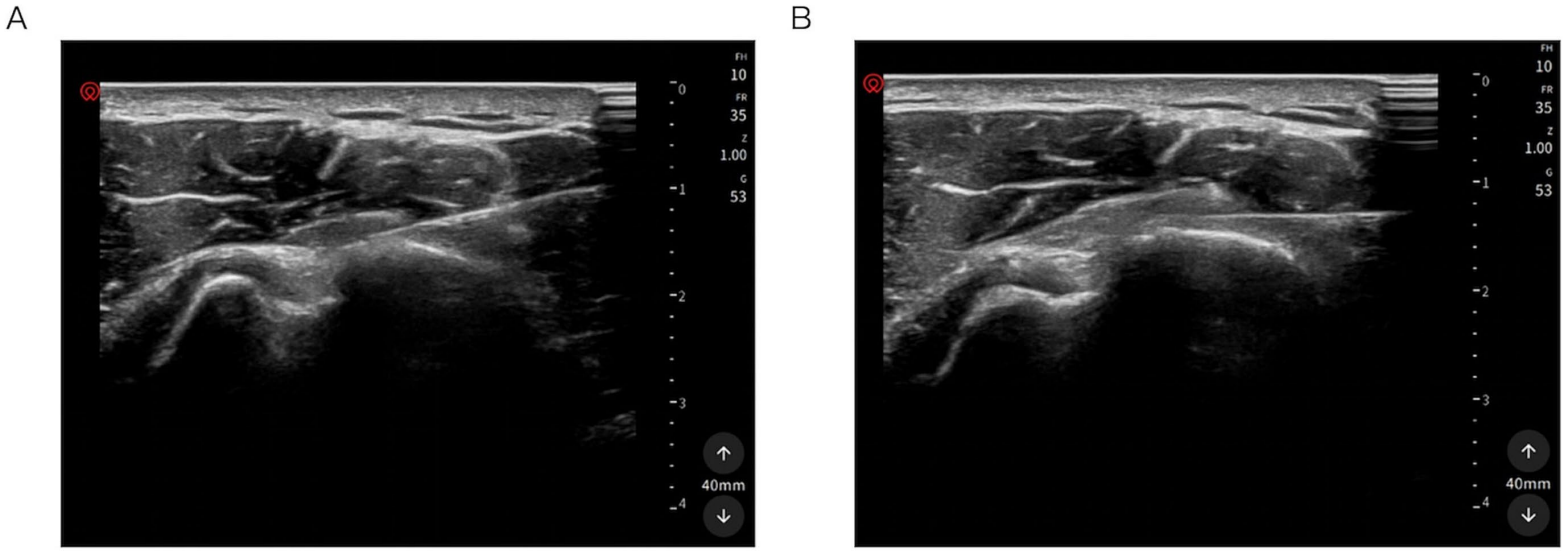
Ultrasound-guided PRP injection. **(A)** Injection with the shoulder in the neutral position. **(B)** External rotation of the shoulder to optimize the spread of the injectate within the injured tendon.

A 2 mL mixture of 2% lidocaine and 0.9% normal saline (1:1 ratio) will be injected using a 25-gauge needle to separate the adherent bursa and anesthetize the rotator cuff tendon. The anesthetic effect will be limited to the needle insertion path and will not extend to the wider area surrounding the target injury region. After the local lidocaine injection, PRP will be administered 1–2 min later to avoid high concentrations of anesthetics remaining in the needle tip channel. To achieve optimum efficacy, the injection strategy will be designed according to MRI findings and clinical signs indicating the origin of the pain. To control pain intensity, a nerve block in the affected area should also be considered. The entire injection procedure will be performed under real-time US guidance to ensure the correct injection pathway and to reconfirm accurate needle placement (Figure 3). After the injection, patients will remain in the supine position under close observation for 15 min.

### 2.4 Study assessments

#### 2.4.1 MRI

All patients will undergo 3.0 T-MRI using the Intera Achieva 3.0T MRI system (Philips), which has a reported sensitivity of 0.98 and specificity of 0.96 for the detection of tendon tears (26, 27). The scanning parameters include a field of view of 22 cm × 22 cm, a matrix size of 256 × 256, and a slice thickness of 3 mm with a 1 mm gap between valid cuts. We will collect T1-weighted and proton density-weighted fat-saturated images (axial, sagittal, and coronal) and T2-weighted and proton density-weighted fat-saturated images (axial, sagittal, and coronal). Sagittal T2-weighted images at the anterior and posterior positions will be used to measure the size of the rotator cuff tear (28). The diagnosis of RCI and the evaluation according to the Ellman classification will be independently performed by two professional radiologists based on MRI findings. Any disagreements will be resolved through consensus (29, 30).

#### 2.4.2 US scanning

As a viable, convenient, and cost-effective alternative to MRI, real-time US has demonstrated high diagnostic accuracy, with reported sensitivity and specificity of 0.84 and 0.89, respectively (31, 32). All patients will undergo five musculoskeletal ultrasonography examinations with Doppler (Wisonic Clover 60) at a frequency of 5–12 MHz, performed by an experienced musculoskeletal US specialist. The diagnostic criteria for musculoskeletal US and the detection of different types of RCI will follow the methodology described by Apostolopoulos et al. (33).

The patient is positioned in a seated posture, and scanning is performed from the medial to the lateral aspect. First, the biceps brachii muscle is localized, followed by the acquisition of a cross-sectional image of the subscapularis tendon. Subsequently, the subscapularis tendon is gradually aligned with the lesser tubercle, and the patient’s shoulder is passively positioned in maximal external rotation. Next, a short-axis view of the supraspinatus tendon is acquired, during which the patient actively performs shoulder abduction and adduction to assess the tendon’s range of motion. Finally, short-axis images of the teres minor and subscapularis tendons are obtained. The supraspinatus and teres minor muscles are traced laterally to evaluate the tendon’s range of motion until the lateral border of the humeral greater tuberosity is no longer visible. Throughout the procedure, the probe orientation is continuously adjusted to optimize imaging quality, and all acquired images are systematically stored.

#### 2.4.3 Adverse events (AEs)

Patients will be instructed to record any discomfort or adverse reactions occurring during the treatment and follow-up periods, regardless of their relation to the intervention. Investigators will evaluate and record each AE with respect to causality (definitely related, probably related, possibly related, possibly unrelated, or definitely unrelated), seriousness, intensity, and expectedness. AEs related to the intervention (definitely, probably, or possibly related) will be defined as treatment-related and included in the primary safety analysis. AEs will be classified by System Organ Class (SOC) according to the latest version of the Medical Dictionary for Regulatory Activities (MedDRAs) and graded from 1 to 5 based on the Common Terminology Criteria for Adverse Events (CTCAEs). All SAEs (grade 4 or 5) will be reported to the Ethics Committee of the Affiliated Hospital of Southwest Medical University and the Chinese Clinical Trial Registry within 24 h via telephone and written email. Safety results will be reported in accordance with the CONSORT Harms statement, including the incidence of total AEs, treatment-related AEs, and SAEs in each group.

The detailed AE classification and reporting framework are presented in the Supplementary file.

#### 2.4.4 Cytokine detection

Peripheral blood samples (5 mL) will be collected from all participants at baseline (2 days before the PRP injection) and at 1, 3, 6, and 12 months after the first PRP injection. The blood samples will be centrifuged at 3000 rpm for 15 min to separate the serum, which will be stored at −80 °C for subsequent analysis. The concentrations of pro-inflammatory cytokines (IL-1β, IL-6, IL-8, IL-10, and TNF-*α*) in serum will be measured using enzyme-linked immunosorbent assay (ELISA) with commercially available kits (R&D Systems, USA) in strict accordance with the manufacturer’s instructions. All measurements will be performed in the central laboratory of the Affiliated Hospital of Southwest Medical University to ensure the accuracy and reproducibility of the results.

### 2.5 Outcome measures

#### 2.5.1 Primary outcome measures

The primary outcomes of this study are shoulder function, assessed using the Chinese version of the Constant-Murley Score (CMS), and pain intensity, assessed using the visual analog scale (VAS), both measured 12 months after the first injection. The Chinese version of the CMS has been validated in the Chinese population, demonstrating good internal consistency (Cronbach’s *α* = 0.739) and test–retest reliability (ICC = 0.827) (34). The Chinese version of the CMS consists of four domains, with a total score ranging from 0 to 100: Pain (1 item, 0–15 points), activities of daily living (ADL, 4 items, 0–20 points), mobility (4 items, 0–40 points), and strength (1 item, 0–25 points). It is a combined self-reported and examiner-based tool, with higher scores indicating better shoulder function (35). Overall shoulder pain will be assessed using the VAS, with scores ranging from 0 (no pain) to 10 (maximal pain). Follow-up time points are summarized in Table 2. Additional details of the outcomes are provided in Table 3.

**Table 2:** Schedules for follow-up assessments and data collection.

Assessments	Baseline (2 days before PRP injection)	End of injection therapy			
		1 month	3 months	6 months	12 months
Chinese version of the CMS	✓	✓	✓	✓	✓
VAS	✓	✓	✓	✓	✓
Tear size (MRI)	✓			✓	✓
US scanning	✓	✓	✓	✓	✓
Cytokine measurements	✓	✓	✓	✓	✓
SF-12	✓	✓	✓	✓	✓
AEs		✓	✓	✓	✓

**Table 3:** Summary of outcomes.

**Category**	**Outcome**	**Description**
Primary outcomes	Chinese version of the CMS	The score consists of four domains: pain (1 item, 0–15), ADL (4 items, 0–20), mobility (4 items, 0–40), and strength (1 item, 0–25), giving a total score ranging from 0 (worst) to 100 (best). Higher scores indicate better function.
	VAS	Overall shoulder pain will be assessed using the VAS, with scores ranging from 0 (no pain) to 10 (maximal pain).
Secondary outcomes	Tear size (MRI) - Ellman classification	Grade I: <3 mm, <25% tendon thickness; Grade II: 3–6 mm, 25–50% tendon thickness; Grade III: >6 mm, >50% tendon thickness.
	US scanning	Shoulder changes will be assessed by US scanning of the rotator cuff tendon integrity, acromioclavicular joints, biceps tendon, posterior labrum, and SASD bursa.
	Cytokine measurements	Changes in cytokine levels (IL-1β, IL-6, IL-8, IL-10, and TNF-α) in peripheral blood.
	SF-12	It is divided into two parts: the Mental Component Summary (MCS) and the Physical Component Summary (PCS). Each component is scored from 0 to 100, with higher scores indicating better quality of life.
	AEs	The most reported AE is post-injection pain, followed by infections. Other AEs include inflammation, allergic reactions, and nodule formation. The detailed AEs classification is displayed in Supplementary file.

#### 2.5.2 Secondary outcome measures

Secondary outcomes include the following: (1) imaging changes assessed by MRI and US (36); (2) serum cytokine levels (IL-1β, IL-6, IL-8, IL-10, and TNF-α); (3) quality of life, assessed using the 12-Item Short Form (SF-12) (37); and (4) AEs (38). Follow-up time points are provided in Table 2. Additional details of the outcomes are provided in Table 3.

### 2.6 Data management

We will collect data from all enrolled participants, including basic demographic characteristics, injection protocol, Chinese version of the CMS scores, VAS scores, MRI findings, US results, SF-12 scores, and AEs, using CRFs. The follow-up duration is 12 months after the first PRP injection, with outcomes assessed according to Table 2. All evaluations will be performed by independent researchers (SJW and XL), blinded to treatment assignment. CRFs will be completed by trained researchers within 24 h of each assessment, following clear instructions to minimize errors. Any missing or inconsistent data will be verified against the original CRFs and resolved through consensus. All data will be encrypted and stored on a secure server to ensure participant confidentiality. Completed CRFs will be reviewed by the trial coordinator (CZ) before data entry. Upon completion of all follow-up time points, CZ will collect all research data and transfer it to a blinded statistician (YXJ) for further analysis.

### 2.7 Sample size

The sample size was calculated based on VAS pain scores, as this outcome exhibits the greatest variability and allows for a conservative estimate. According to a previous randomized controlled trial in patients with partial-thickness rotator cuff tears (20), the minimal clinically important difference (MCID) for the VAS is 1.4 cm, with a standard deviation (SD) of 2.41 cm. To detect this difference between the two groups at 12-month post-injection, with a two-sided significance level of *α* = 0.025 (Bonferroni-adjusted for two primary endpoints) and a power of 80% (*β* = 0.20), the required sample size per group was calculated using the formula for two independent means:


$$n=\frac{2\times {\left(Z_{\alpha /2}+Z_{\beta }\right)}^{2}\times \sigma^{2}}{\delta^{2}}$$

whereZα/2=2.24(forα/2=0.0125),Zβ=0.84(forβ=0.20),σ=2.41,andδ=1.4.This yields:

$$\begin{array}{c} n=\frac{2\times {\left(2.24+0.84\right)}^{2}\times {\left(2.41\right)}^{2}}{{\left(1.4\right)}^{2}}=\frac{2\times {\left(3.08\right)}^{2}\times 5.8081}{1.96}\\ =\frac{2\times 9.4864\times 5.8081}{1.96}\approx 56.2 \end{array}$$

Therefore, 57 participants per group are required. Assuming a 20% dropout rate over the 12-month follow-up period, the final sample size is increased to 72 participants per group, resulting in a total of 144 participants.

The study is also adequately powered for the Chinese version of the CMS, the second primary outcome. Previous studies in similar populations report an SD of approximately 15–20 points and an MCID of approximately 10 points for the CMS (35, 39). With 57 participants per group, *α* = 0.025, and 80% power, the detectable difference is calculated as follows:


$$\delta =(Z_{\alpha /2}+Z_{\beta })\times \sigma \times \sqrt{\frac{2}{n}}=(2.24+0.84)\times \sigma \times \sqrt{\frac{2}{57}}=3.08\times \sigma \times 0.187$$

With *σ* = 18, *δ* is approximately 10.4 points, and with *σ* = 15, *δ* is approximately 8.6 points. Both values are within or below the established MCID range, confirming that the sample size is sufficient to detect clinically meaningful differences in the CMS.

For secondary and exploratory outcomes (e.g., MRI findings), the study is not specifically powered. These analyses will be considered hypothesis-generating, and the results should be interpreted with caution.

### 2.8 Statistical analysis

Statistical analyses will be performed using SPSS 25.0 (IBM Corporation, Armonk, NY, USA) and R 4.0 (R Foundation for Statistical Computing, Vienna, Austria) by an independent statistician (YXJ) who will remain blinded to treatment allocation until all primary analyses are completed. The intention-to-treat (ITT) principle will be applied to all primary analyses, including all randomized participants who will receive at least one PRP injection. A per-protocol (PP) analysis will be conducted as a sensitivity analysis, excluding participants with major protocol violations.

#### 2.8.1 Descriptive statistics

Continuous variables will be summarized as mean ± SD or median with interquartile range (IQR). Categorical variables will be presented as frequencies and percentages (*n* %). Normality will be assessed using the Shapiro–Wilk test, and homogeneity of variance will be examined with Levene’s test.

#### 2.8.2 Baseline comparability

Baseline characteristics will be compared between the groups using independent-samples *t*-tests or the Mann–Whitney *U*-test for continuous variables and chi-squared tests or Fisher’s exact test for categorical variables. Variables showing between-group differences at a *p*-value of <0.10 will be included as covariates in adjusted analyses.

#### 2.8.3 Primary outcome analysis

The primary outcomes (the Chinese version of the CMS and the VAS at 12 months) will be analyzed using analysis of covariance (ANCOVA), with treatment group as a fixed factor and baseline score, age, and time from diagnosis to injection as covariates. A Bonferroni-corrected significance level of *p* < 0.025 (two-tailed) will be applied to each primary outcome to account for multiple comparisons. The study will be considered positive if at least one primary outcome achieves statistical significance at the adjusted α level, provided that the other outcome shows consistent directional improvement.

#### 2.8.4 Secondary and exploratory outcomes

MRI and US findings will be compared using Fisher’s exact test or ordinal logistic regression (adjusted for baseline severity and age). Longitudinal changes in continuous parameters will be analyzed using linear mixed-effects models (LMMs). The SF-12 scores at 12 months will be compared using *t*-tests or the Mann–Whitney *U*-test, with LMMs applied to assess longitudinal profiles. Cytokine levels will be log-transformed if necessary and analyzed using two-way repeated measures ANOVA or LMMs. Correlations between cytokine changes and clinical outcomes will be assessed using Spearman’s rank correlation coefficient. AEs will be tabulated by group and severity (CTCAE grade). The incidence of total AEs, treatment-related AEs, and SAEs will be compared using the Fisher’s exact test.

#### 2.8.5 Longitudinal analysis

For repeated measurements at baseline and at 1, 3, 6, and 12 months, LMMs will be used, including fixed effects for group, time, and the group-by-time interaction, with a random intercept for each participant. An unstructured covariance structure will be specified, and the Kenward–Roger approximation will be used to calculate the denominator degrees of freedom. Age will be included to explore age-by-treatment interactions; if the interaction is significant (*p* < 0.10), stratified analyses will be performed.

#### 2.8.6 Subgroup analyses

Prespecified subgroup analyses for the primary outcomes will be conducted based on age (18–45 years vs. 46–65 years), Ellman classification (Grade I/II vs. Grade III), and time from injury to injection (0–2 weeks vs. 2–4 weeks) to ensure the balance of key confounding factors between the two groups. Interaction tests will be performed within LMMs, and all subgroup analyses will be considered exploratory.

#### 2.8.7 Handling of missing data

Missing data will be assumed to be missing at random (MAR). Primary analyses will be conducted using LMMs, which are robust to MAR and utilize all available data. For ANCOVA at 12 months, multiple imputation by chained equations (20 imputations) will be performed using the R package ‘mice,’ including all baseline covariates and outcome values at other time points. Results will be combined using Rubin’s rules. Sensitivity analyses will include complete-case analysis and last observation carried forward (LOCF) imputation.

#### 2.8.8 Safety analysis

Safety will be assessed for all participants receiving at least one PRP injection. AEs will be coded using MedDRAs and summarized by SOC and Preferred Term (PT). Descriptive statistics and 95% confidence intervals will be reported; no formal hypothesis testing is planned for safety endpoints.

## 3 Discussion

Tendons are bundles of parallel fibrous structures that lack blood supply, making them difficult to heal if damaged. After a tendon laceration or rupture, the tissue is often repaired by scar formation, which cannot restore the tendon’s normal mechanical structure. PRP, an emerging biological treatment, has been widely applied to the management of tendinopathy. However, heterogeneity in PRP preparation and variability in its cellular composition fuels ongoing controversy over its clinical efficacy. This study has been designed to investigate the role of leukocytes and PLTs in PRP for tendinopathy. By comparing two distinct PRP formulations, this study will provide critical evidence to guide the application of optimal PRP composition for tendinopathy.

It is imperative to consider the 5R’s of prescribing: “right patient, right drug, right dose, right route, and right time.”

Patient characteristics may be the factor directly affecting the quantity of PRP. When evaluating the quality of PRP, it is necessary to consider patient-related variables that could affect its composition, such as female sex, age over 50 years, traumatic clinical conditions, or a history of medications such as clopidogrel and dabigatran (40). The biological status of the tendon determines the clinical effects of PRP (15). Conservative treatment can be the first option after evaluating related risk factors that may lead to failure of non-operative management (29, 41). When non-operative management fails, surgical treatment remains the optimal approach for RCI with significant symptoms, especially articular-side tears exceeding 6 mm of the rotator cuff footprint or complete tears (29, 42). Therefore, choosing the “right patient” is essential in clinical practice.

A standardized preparation procedure is an important factor influencing clinical efficacy, as it has a direct impact on the final composition and quality of PRP. PRP can be formulated in multiple ways, and there is currently no internationally accepted consensus to standardize its formulation. It seems that the concentration of PRP components produced by the machine is relatively stable. In fact, the concentrations of PLTs, leukocytes, and GFs may vary depending on the equipment used. Several factors can affect the quality of PRP products, including anticoagulant type, g-force, centrifugation duration, cell volume, and separation accuracy (40). To achieve optimal clinical effects, PRP components and doses should be tailored to the specific scenario. In this study, the PLT concentration for Group 1 is set at 2.5–3.5-fold enrichment (1.74 ± 0.20 × 10^9^/2 mL) and for Group 2 at 4.0–6.0-fold enrichment (2.66 ± 0.52 × 10^9^/2 mL), administered once per week for two consecutive weeks. Compared to higher concentrations with doses of 4–6 mL used in knee osteoarthritis, the PRP dose for tendinopathy is usually set at 2–3 mL, as the injection strategy focuses on the injured or degenerated tendons rather than the intra-articular space. This dosage scheme is based on the anatomical characteristics of the shoulder joint and clinical practice of PRP for RCI. It has been reported that a 3–5-fold enrichment of PLTs is recommended as the optimal concentration for tendinopathy (43). Higher concentrations of PRP may lead to tendon overgrowth and even cause shoulder impingement syndrome (44). Excessively high PRP concentrations may even have an inhibitory effect on cell proliferation, migration, and the production of type I collagen (45). As an important part of tissue healing, inflammatory cells such as neutrophils and monocytes migrate to the injured area and remove necrotic tissue through phagocytosis (15). The leukocyte concentration plays a crucial role in PRP function, influencing both anabolic and catabolic processes, which are often time-dependent (46). LR-PRP can induce a pronounced inflammatory response, increasing cytokine levels such as IL-6, IL-10, and TNF-α, especially TNF-α, which inhibits apoptotic cell death by activating the NF-kB signaling pathway. Therefore, LR-PRP seems to have a more effective regeneration tendency in tendon healing at the early stage (43). In contrast, LP-PRP can improve the biomechanical properties of tendons, including tensile stress and stiffness, due to its beneficial effects on tendon proliferation, collagen synthesis, and inflammation modulation (15, 47). However, in the application of LP-PRP, this approach may be more suitable for chronic tendinopathy (48). PRP-related approaches should be further refined in terms of preparation parameters to achieve the “right drug,” tailored to specific clinical indications and usage.

The unstandardized timing of PRP injections also hinders the evaluation of efficacy across different studies. Although previous studies have investigated different types of PRP in acute or chronic tendon injury phases, the optimal intervention time points remain uncertain (3, 15, 40, 46). The specific PRP formulation should align with the tissue healing time point (49). For example, the natural healing process can be delayed or interrupted by PRP injections during the acute inflammatory phase, as the dual pro-and anti-inflammatory properties of PRP may perturb the orderly inflammatory response (49).

The objective of PRP injection is to minimize pain and reduce the risk of tendon tears. The specific site of the PRP injection is a key factor influencing its efficacy. Pain-generating areas are defined as areas with a high density of nociceptive receptors, including the subacromial bursa, coracoacromial ligament, and the long head of the biceps tendon. These areas are innervated by the same nerves—the suprascapular, lateral pectoral, and axillary nerves—that also innervate the injured rotator cuff. Shoulder pain originating from RCI can be transmitted to distinct pain generator zones via three neural bridges (50). Consequently, when designing injection strategies and performing selective nerve blocks, clinicians should target not only the injured tendon but also the pain generator areas and their parent nerve trunks to discrepancies in efficacy. The injection procedure can ensure precise delivery of PRP to the injured site under US guidance.

Most previous studies investigated the role of leukocytes or PLTs in clinical practice. Based on current research, this study will first investigate the synergistic effects of PRP components and their concentrations, and will subsequently analyze the relationships between these factors and patient variables in a stepwise manner. This protocol reports a detailed preparation method and PRP characteristics, aiming to stimulate discussion on the optimal PRP formulations for different musculoskeletal conditions and to promote the development of standardized PRP guidelines in the future. Although this study focuses on several factors affecting the practical efficacy of PRP, many uncertainties may still exist in its clinical application, which need to be reported and investigated in further studies. Future studies should expand the sample size, extend the follow-up period, and include multi-center data to validate the results. In addition, further *in vitro* experiments are needed to explore the specific mechanism underlying the synergistic effects of PLT and leukocyte concentrations on rotator cuff tendon healing.

## 4 Conclusion

PRP composition has a close relationship with clinical efficacy and should be tailored to specific clinical characteristics. To inform clinical practice, all relevant factors should be considered collectively when reporting PRP outcomes.
