# Supplementary material for: Comparison of two types of platelet-rich plasma in rotator cuff injury: study protocol for a randomized clinical trial
Source: Front Med (Lausanne). 2026 Mar 13;13:1791881. doi: 10.3389/fmed.2026.1791881 (PMC13022979; doi:10.3389/fmed.2026.1791881)
Supplement: Supplementary file 1 [file Data_Sheet_1.pdf]

Supplementary File

1 The detailed AEs classification and reporting framework

1.1 Grades of PRP Injection-Related Specific AEs

The severity of all AEs is uniformly graded according to the Common Terminology Criteria for Adverse Events (CTCAE), the global gold standard for clinical trial AE grading, which classifies all events into 5 grades based on clinical symptoms, intervention requirements, and impact on daily activities and study participation. The PRP injection-specific AE grading details (adapted to rotator cuff injury treatment) are as follows.

Table 1 PRP Injection-Related Specific AEs classification

Grade	PRP Injection-Related Specific AEs
1: Mild	1. Mild injection site reaction: Local tenderness, slight erythema/swelling (range <1cm) without obvious pain, self-relieving within 1-2 days without intervention.<br>2. Mild shoulder pain: Transient soreness at the injection site, no impact on shoulder joint activity, no need for analgesics.<br>3. Mild skin reaction: Local slight pruritus without rash, no medication required.
2: Moderate	1. Moderate injection site reaction: Local erythema, swelling, and tenderness (range 1-2cm), requiring oral non-steroidal anti-inflammatory drugs (NSAIDs) for symptom relief.<br>2. Moderate shoulder activity limitation: Mild limitation of shoulder abduction/rotation, affecting minor daily activities but not basic living, relieved by local hot compress/physiotherapy.<br>3.Moderate allergic reaction: Local urticaria/rash, requiring oral antihistamines for intervention.
3: Severe	1. Severe injection site reaction: Severe local redness, swelling, and induration (range ≥2cm), accompanied by persistent severe pain, requiring intravenous anti-inflammatory/analgesic drugs or local puncture aspiration.<br>2. Severe shoulder dysfunction: Obvious limitation of shoulder joint activity (abduction <90°), unable to complete daily activities (dressing, combing hair), requiring inpatient physical therapy/specialized intervention.<br>3. Local infection: Inflammatory exudate at the injection site, requiring intravenous antibiotics for anti-infection treatment.<br>4. Severe allergic reaction: Widespread rash over the body, mild laryngeal edema with no dyspnea, requiring intravenous anti-allergic drugs.
4: Life-threatening	1. Severe systemic infection: Local infection progresses to sepsis/septic shock, requiring ICU monitoring and anti-shock/anti-infection rescue.<br>2. Severe allergic reaction: Anaphylactic shock, severe laryngeal edema with dyspnea/suffocation, requiring adrenaline injection, tracheal intubation, or emergency tracheotomy.<br>3. Severe bleeding: Joint cavity massive hemorrhage or systemic coagulation dysfunction due to PRP injection, threatening life and requiring emergency hemostasis/surgical intervention.<br>4. Complete shoulder joint immobilization: Severe adhesion of the shoulder joint leading to complete loss of activity, requiring emergency surgical release.
5: Fatal	Death caused by TRAEs such as severe anaphylactic shock, uncontrolled sepsis/septic

	shock, or severe intracranial/joint bleeding induced by PRP injection.

1.2 Standardized reporting and management

This study strictly follows the CONSORT Harms statement and international clinical trial AE reporting norms for standardized AE management and reporting:

1.2.1 Routine reporting: All non-serious AEs (Grade 1-3) are recorded in detail, including occurrence time, grade, causality, intervention measures, and outcome, and are summarized and reported monthly to the study supervision group.

1.2.2 Serious adverse event (SAE) rapid reporting: All Grade 4 and 5 AEs are defined as SAEs. Investigators must complete the SAE reporting form within 24 hours of discovering an SAE and report it to the Institutional Review Board (IRB) of the Affiliated Hospital of Southwest Medical University, Chinese Clinical Trial Registry, and study sponsor via telephone and written email simultaneously. The report includes detailed clinical data, emergency treatment measures, and preliminary causality assessment.

1.2.3 Follow-up of AEs: All AEs are followed up until complete remission, stable condition, or a definite outcome is reached; for AEs with persistent sequelae, long-term follow-up and outcome assessment are conducted until the end of the study.

1.2.4 Blinding maintenance for SAE: In the event of an SAE requiring unblinding for clinical treatment, the independent non-blinded researcher is responsible for single patient unblinding, and the unblinding information is kept confidential and not disclosed to other study personnel to ensure the overall blinding of the trial is maintained.

All AE data will be statistically analyzed by an independent biostatistician who is blinded to the treatment allocation, including the incidence of total AEs, TRAEs, SAEs, and the distribution of AE grades and system organ classifications (SOC) in the two groups, to comprehensively evaluate the safety of the two PRP formulations.
